# Core Needle Biopsy Guidance Based on Tissue Morphology Assessment with AI-OCT Imaging

**DOI:** 10.3390/diagnostics13132276

**Published:** 2023-07-05

**Authors:** Gopi Maguluri, John Grimble, Aliana Caron, Ge Zhu, Savitri Krishnamurthy, Amanda McWatters, Gillian Beamer, Seung-Yi Lee, Nicusor Iftimia

**Affiliations:** 1 Physical Sciences Inc., Andover, MA 01810, USA; gmaguluri@psicorp.com (G.M.); jgrimble@psicorp.com (J.G.); acaron@psicorp.com (A.C.); gzhu@psicorp.com (G.Z.); 2 MD Anderson Cancer Center, Houston, TX 77030, USA; skrishna@mdanderson.org (S.K.); amcwatters@mdanderson.org (A.M.); 3 Aiforia Inc., Cambridge, MA 02142, USA; gillian.beamer@aiforia.com (G.B.); seungyi.lee@aiforia.com (S.-Y.L.)

**Keywords:** tissue biopsy guidance, optical coherence tomography imaging, artificial intelligence

## Abstract

This paper presents a combined optical imaging/artificial intelligence (OI/AI) technique for the real-time analysis of tissue morphology at the tip of the biopsy needle, prior to collecting a biopsy specimen. This is an important clinical problem as up to 40% of collected biopsy cores provide low diagnostic value due to high adipose or necrotic content. Micron-scale-resolution optical coherence tomography (OCT) images can be collected with a minimally invasive needle probe and automatically analyzed using a computer neural network (CNN)-based AI software. The results can be conveyed to the clinician in real time and used to select the biopsy location more adequately. This technology was evaluated on a rabbit model of cancer. OCT images were collected with a hand-held custom-made OCT probe. Annotated OCT images were used as ground truth for AI algorithm training. The overall performance of the AI model was very close to that of the humans performing the same classification tasks. Specifically, tissue segmentation was excellent (~99% accuracy) and provided segmentation that closely mimicked the ground truth provided by the human annotations, while over 84% correlation accuracy was obtained for tumor and non-tumor classification.

## 1. Introduction

Percutaneous biopsy has been established as a safe, effective procedure for cancer diagnosis. The success rate of the biopsy is measured by the ability to collect sufficient viable material for molecular genetical and histological analysis [1,2]. However, due to the heterogeneity of the tumor tissue, biopsy sensitivity/specificity varies within a relatively large range (65% to 95%) [3,4,5,6]. Therefore, proper biopsy guidance has become a real clinical need.

Although radiologic imaging is used to guide biopsy needle placement within the tumor, it does not provide sufficient resolution to assess tissue cellularity, which is defined as the ratio between viable tumors and benign stroma constituents. For example, high-resolution ultrasound (US) has been used to provide biopsy guidance, but still does not provide the expected results as its resolution is not sufficient to resolve tissue morphology at the micron scale, which is needed to properly assess its cellularity [7,8]. US is both operator-dependent and needs a radiologist experienced in sonography to correctly interpret imaging findings [9,10]. 

Without proper guidance, biopsy often needs to be repeated, leading to significant cost to the health care system [11,12]. Considering that millions of core needle biopsies are performed annually in the US [13,14,15], if on average 20% of these procedures need to be repeated [12], the additional costs to the health care system become immense. 

Besides the financial implications, the inadequate quality of the biopsy specimens can have a negative impact on downstream molecular pathology and can delay pathway-specific targeted therapy [16]. Furthermore, as novel therapeutics are routinely introduced with companion biomarkers, biomarker testing is expected to become the standard of care in the very near future. Towards this end, FDA has mandated that targeted therapies shall be accompanied by patient-tailored companion diagnostic tests [17,18]. As a result, it is envisioned that image-guided biopsies will start playing a significant role in oncologic clinical trials. Thus, techniques able to provide the reliable assessment of tissue at the cellular scale, at the time of sampling, will be essential to reliably obtain adequate amounts of viable tumor tissue for biomarker analysis. Biopsy cores with large amounts of necrotic or non-tumor tissue are not suitable for such tests. 

Various optical technologies have been explored to guide biopsy and improve biopsy sampling. Such technologies include Raman spectroscopy, dynamic light scattering, optical coherence tomography (OCT), etc. [19,20,21,22,23,24,25,26,27,28]. Among them, OCT has shown significant promise due to its ability to assess true tissue morphology within relatively large volumes of tissue, such as the size of the biopsy cores, at a higher speed than of the other modalities. OCT is routinely used for differentiating between normal tissue and cancer in various organs [19,20]. However, the interpretation of the OCT images can be highly subjective, as the readers can have a different understanding of the tissue morphology shown by the images. Furthermore, when performing a biopsy procedure, the interventional radiologist must decide about the biopsy location in real time. Therefore, we investigated using user-assisted deep learning (DL), a subset of artificial intelligence (AI) based on deep neural networks, for rapid image analysis. AI has made remarkable breakthroughs in medical imaging, especially for image classification and pattern recognition [29,30,31,32,33,34,35]. Studies showed that OCT image evaluation by DL algorithms has achieved good performance for disease detection, prognosis prediction, and image quality control, suggesting that the use of DL technology could potentially enhance the efficiency of the clinical workflow [36,37]. 

This paper presents a novel AI-OCT approach for the real-time guidance of core needle biopsy procedures. A hand-held OCT probe was developed to collect in vivo images from a rabbit model of cancer. Selected OCT images were used to train an AI model for tissue type differentiation. The images were selected based on the pathologist’s feedback, with annotated normal and tumor tissue areas. The performance of the AI model was assessed against the annotations performed by trained OCT image readers. The AI model showed similar results to those of the humans performing the same classification tasks. Specifically, tissue boundary segmentation was excellent (>99% accuracy) as it provided segmentation results that closely mimicked the ground truth provided by the human annotations, while >84% correlation was obtained for tumor and non-tumor classification.

## 2. Materials and Methods

**Figure 1:**
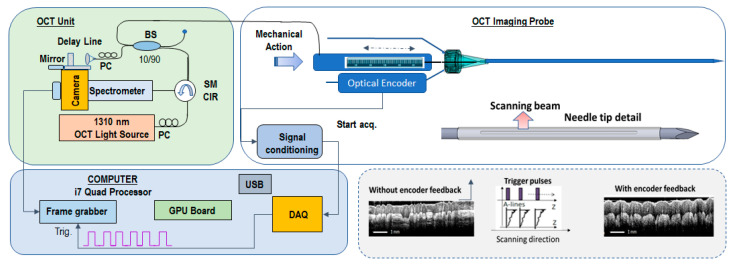
OCT imaging scheme based on encoder-triggered A-line acquisition.

*OCT Instrumentation*: A customized OCT imaging approach, previously reported by our team [38], was used in this study. In brief, using this approach, an axial OCT reflectivity profile (also called an A-line) is acquired only when an incremental movement of the OCT catheter probe is detected by a linear encoder (see the concept in Figure 1). The encoder creates a trigger that is sent to a data acquisition (DAQ) card. The DAQ card initiates the data acquisition and processing sequence. Each processed OCT signal (A-scan) is inserted into an array that is appended at each incoming encoder trigger to form an OCT image. 

By using this approach, data collection can be performed at variable speeds of the probe advancement through the tissue, enabling the use of an either manual or motorized scanning approach for the OCT catheter. Scanning linearity does not impact image quality.

The OCT instrument, based on the spectral domain approach, uses a 1310 nm light source with a bandwidth of approximately 85 nm, providing an axial resolution of ~10 um, which supports the detection of small tissue features at the cellular level. The light from the broadband source is split into the sample and reference arms of the interferometer by a 10/90 fiber splitter. A fiber optic circulator is inserted between the light source and fiber splitter to maximize the return from both arms of the fiber interferometer. The interference signal obtained by mixing the returned light from the sample with that from the reference arm is sent to a linear array camera. The fringe signals are digitized by a camera link frame grabber and processed in real time with a graphical processing unit (GPU).

**Figure 2:**
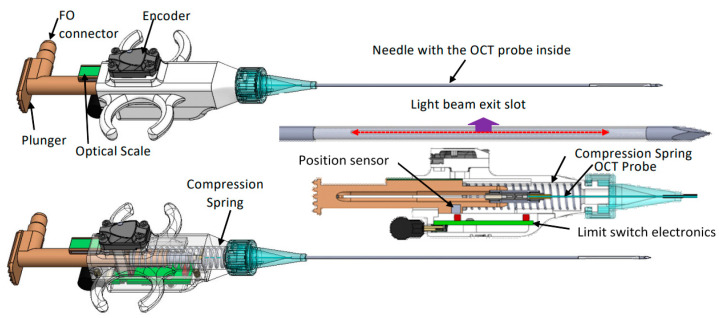
CAD design of the biopsy probe. **Top**—general view. **Middle**—needle and spring-loaded mechanism details. **Bottom**—transparent view.

*OCT probe*: A specially designed OCT probe, suitable for tissue investigation through the bore of the biopsy needle, was used in this study. A simplified schematic design of this probe is shown in Figure 2. 

As observed, the probe consists of four major parts: probe main body, plunger, encoder, and a needle containing the OCT fiber optic catheter. The plunger is spring-loaded and has the fiber optic OCT catheter attached to it through a fiber connector (see cross-sectional and transparent views). When pressed, the plunger moves the OCT catheter forward within a custom-made needle. The OCT light exits the needle through a slot made at the tip of the needle. The needle is covered on the slot area by a fluorinated ethylene propylene (FEP) tube to seal the OCT catheter inside the needle and prevent the tissue from catching on the needle. To secure the FEP tube in place, the needle is slightly tapered towards its tip, where the axial slot is located. An optical encoder is attached to the probe holder and used to monitor the movement of the OCT fiber optic catheter relative to an optical scale, which is also attached to the plunger. A sliding mechanism is used to maintain the scale parallel to the encoder surface, such that correct scale readings are provided during the relative movements of the OCT catheter to the main body of the hand-held probe.

The custom-made needle is attached to the probe holder through a luer-lock cap, identical to that of commercial syringes. As a result, this needle can be easily replaced during the procedure, if needed. An electronic circuit inserted into the probe body is used in an A-line acquisition only when the plunger is moved over a distance of at least 1 mm. Thus, it blocks false triggers generated by the small vibrations during probe insertion within the tissue, before pushing the plunger. This circuit also formats the trigger signal, so it can be reliably sent through a 2 m length mini-USB cable to the instrumentation unit. 

The OCT fiber catheter consists of a single mode (SM) fiber, terminated with a micro lens, polished at 45 deg, to send the light orthogonally to the catheter scanning direction. The catheter is encapsulated within a 460 um outer diameter hypodermic tube, terminated with a fiber optic connector (Model DMI, Diamond USA).

**Figure 3:**
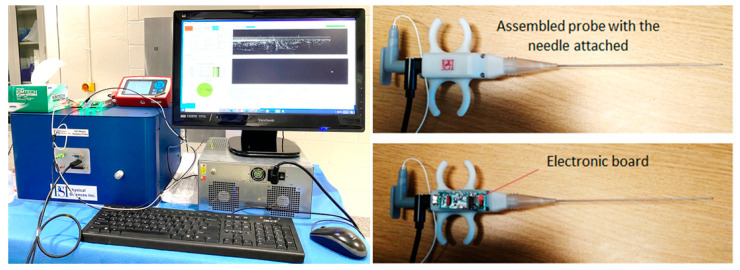
Photographs of the OCT instrument and OCT probe.

Photographs of the Gen I instrument and biopsy guidance probe are shown in Figure 3. The instrumentation rack is small (16″ × 14″ × 12″) and incorporates the power supply, the spectrometer, the optical delay line, the light source, and the fiber optic spectrometer. The computer can be placed on the side or underneath. The instrumentation unit can be placed within a commercially available wheeled rack to add portability. The OCT probe is easy to use: the plunger can be pushed with the thumb, while the index and the middle fingers can be inserted through probe ears to hold it in place. OCT images at multiple angular positions can be generated by successively rotating the probe, while still in the tissue, and repeating the scans of the OCT catheter. 

*Animal model*: A rabbit model of cancer—the Albino New Zealand White (NXW) Rabbit, Strain Code 052—was used to perform an in vivo study for technology evaluation at MD Anderson Cancer Center (MDACC), Houston, TX, USA. All experiments were performed in agreement with the MDACC IAUCUC approved animal protocol—00001349-RN00-AR002. 

A total of 30 animals were prepared for this study using the following protocol:

(a) Percutaneously, intramuscularly inject VX2 tumor in both thighs of each rabbit;
(b) Allow tumor to grow for 10 to 14 days +/− 2 days to reach a size of 1.5 to 2 cm in diameter (appropriate size for use);
(c) Use palpation to verify tumor growth in thighs and determine tumor growth and volume.

*Data collection:* The imaging protocol included the next steps:

- Percutaneously insert a biopsy guidance needle (18 Ga) within the tumor using ultrasound guidance;
- Remove the needle stylet and insert the optical probe into the tumor site through the bore of the guidance needle;
- Perform up to 4 quadrant OCT measurements (4 × 90 deg angular orientations) at each location and collect at least 2 images/quadrant;
- Retract the OCT probe and use an 18 Ga core biopsy gun to collect 1 biopsy core after imaging is performed;
- Reinsert the guidance needle in the tumor-adjacent area and repeat the steps above to collect OCT images of heathy tissue;
- Following the final biopsy, euthanize the animal using Beuthanasia-D (1 mL/10 lb) solution.

**Figure 4:**
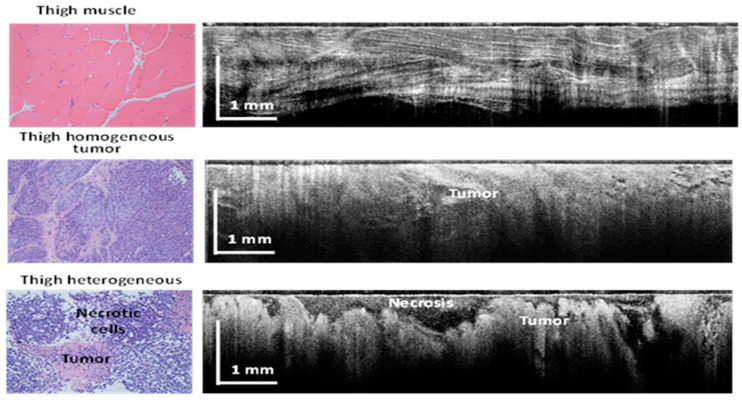
Examples of OCT-collected images and associated pathology for each tissue type.

As each animal had 2 tumor inoculations (one in each thigh), with a minimum of 4 images collected from each site, plus one image of the healthy tissue near each tumor site, over 300 images were collected. Representative examples of such images are shown in Figure 4. As can be easily observed, morphology details, such as fiber muscle bundles and micro vessels, were well recovered by OCT. Approximately 100 images corresponding to each tissue type were selected for the AI algorithm training set. These images were selected in collaboration with the pathologist to best match the pathology findings. 

*Data Processing:* OCT image analysis was performed using Convolutional Neural Network (CNN) artificial intelligence (AI) software, Aiforia Technologies Oyj, Pursimiehenkatu 29-31, FI-00150 Helsinki, Finland. This is a supervised deep learning software for image analysis, which uses annotated data for AI algorithm training. The AI model was designed to segment tissue boundaries, while excluding air–tissue interfaces and surface artifacts (e.g., vertical white lines, “shadows” from blood vessels), and then to segment 3 regions of interest within the tissue: cancer (tumor); necrosis within the tumor; and healthy, also referred to here as “non-tumor”. 

The AI model was structured in 3 layers/3 classes, as shown in Table 1. The main goal was to differentiate between normal and cancer tissue. The first class, called “Tissue”, defines tissue boundaries (top and bottom). Once boundaries are detected, the next step is to differentiate the two major classes: non-tumor or healthy tissue, and tumor tissue. The tumor class contains a sub-layer called necrotic tissue. This is an important subclass to be highlighted, as the necrotic tissue does not provide any diagnostic value. 

CNN1 is a parent layer and CNN2 and CNN3 are child layers. They are independent layers connected to each other by filtering. The model training parameters and image augmentation parameters for each class are shown in Table 2.

**Figure 5:**
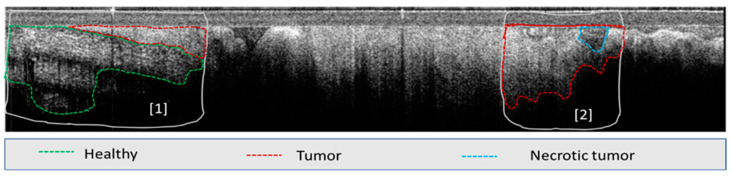
Example of OCT image annotation: see area (1) and area (2).

A total of ~100 OCT images with 4.2 μm/px resolution were selected for the initial training of the AI model. The images were in bmp format with 2400 × 600 pixels, corresponding to an area of 2.5 mm × 10 mm. Most of the images contained one single tissue type, except for the necrotic tissue, which is inherently present within the tumor. As this relatively low number of images has been proven to not provide satisfactory results, additional images were added for model training. However, since the remaining images contained more than one tissue type, they were annotated by expert OCT image readers to define the boundaries of each tissue type (see example Figure 5). The selection of multiple areas in each image enabled a significant increase (~3×) in the total training set of images.

Although the total number of training images was still relatively small for an AI model, the model was able to produce satisfactory results by using a supervised training approach. OCT reader supervision was used during AI software development to optimize the model for the available training data. Over 100 selected regions were visually inspected by our team during AI model development to determine if tissue boundaries were properly detected, or if the cancer/normal tissue interface was properly differentiated. OCT reader supervision greatly improved the AI model performance. 

## 3. Results

After careful training, the AI model was applied to a validation set of ~100 images, not included in the training set. The AI results were compared against OCT image reader annotated images. The results were quite satisfactory, considering the relatively small training set of images used in this preliminary evaluation.

**Figure 6:**
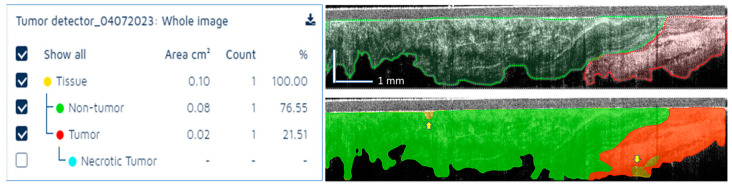
Example of tissue segmentation using the AI model. **Left**—AI summary of tissue area for each tissue class. **Top right**—OCT reader annotated image. **Bottom right**—AI segmentation results. **Green**: Normal tissue; **Red**: Tumor tissue.

A representative example of tissue differentiation by the classes specified above is shown in Figure 6. As it may be observed, a few areas were not classified (see yellow arrows), as the AI model was not able to associate the tissue to a specific class with high certainty (>90%). Overall, the OCT reader–AI agreement was good. The bottom boundary was more accurately identified by the OCT reader, while the AI model slightly overestimated tissue depth in some locations. 

**Figure 7:**
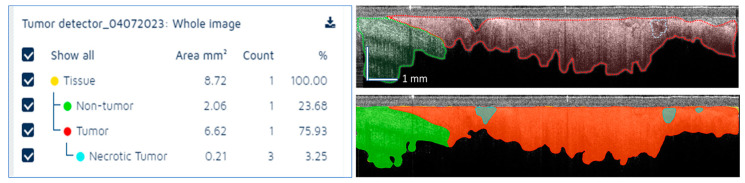
Example of tissue segmentation using the AI model. **Left**—AI summary of tissue area for each tissue class. **Top right**—OCT reader annotated image. **Bottom right**—AI segmentation results. **Green**: Normal tissue; **Red**: Tumor tissue; **Blue**: Necrotic tissue.

Another representative example is shown in Figure 7, where cancer tissue is present in a larger amount than the normal tissue, indicating that this area is appropriate for taking a biopsy core. Very small areas of potential necrosis were detected; however, this will not be a real concern for the interventional radiologist, as the amount of tumor tissue is fairly large (over 75% of the scanned area).

The entire validation set of images was analyzed by three OCT readers who individually made the annotations and the consensus among readers was analyzed. Areas of each tissue type were calculated for each reader, as well as for the AI-segmented images. Reader agreement (human vs. human), as well as the AI vs. human agreement, was analyzed. The false positive (FP) rate, false negative (FN) rate, precision, sensitivity, and F1 score were assessed for each class, using the formulas defined in Table 3. 

The accuracy parameters were calculated for the validation set of images, as summarized in Table 4, Table 5, Table 6 and Table 7.

As may be observed, false negatives, false positives and errors were under 3% for normal and tumor tissue, while the error was somewhat higher (~5%) for necrotic tissue. The precision, the sensitivity, and the F1 score were within the 70% range for the normal and tumor for both AI vs. human and human vs. human. However, lower values were obtained for necrotic tissue. It is to be noted that the F1 score is the preferred metric for evaluating the accuracy of the AI model. It combines the precision and recall scores of a model. This accuracy metric computes how many times a model made a correct prediction across the entire dataset.

The AI model vs. human and human vs. human agreement was calculated as a fraction using the following formula:

Agreement [%] = 100 − (|AI vs. human % parameter − Human vs. human % parameter|) (1)

Over 97% agreement between AI and human findings was obtained for the F1 score, while somewhat lower agreement (~84%) was obtained for necrotic tissue. 

## 4. Discussion

The AI-OCT technology was preliminarily evaluated on an animal model of cancer to determine its feasibility for safe in vivo use, while the potential use of the AI approach for the real-time assessment of tissue composition at the tip of the biopsy needle was analyzed as well. 

The proposed encoder feedback approach has been proven to work reliably and generate high-quality micron-scale images with a rate of 1–2 images/s, mainly dictated by the user ability to perform a faster or slower mechanical scan of the OCT probe by pushing/releasing the probe plunger. In some cases, motion artifacts were noted if the user did not have a steady hand and the probe was moved while acquiring an OCT scan. Therefore, further implementation will consider the use of a motorized probe. 

The AI model was optimized for the current training OCT dataset, which used 255 images. It was noted that there were regions within the tissue where the model could not accurately classify as tumor or non-tumor regions. There are likely two related reasons: First, these regions also made it challenging for human annotators and ground truth experts to agree upon the class designation and make accurate annotations for model training. This is because the visual patterns in the shades of white, gray, and black that humans recognize as “tumor” or “non-tumor” in some regions of the OCT images overlap in their morphology. Second, the number of images in the training dataset with these types of challenging regions was relatively small. Certainly, it is possible to substantially improve model classification accuracy with additional training data. Therefore, this is the next step we propose to take to further evaluate the potential of the AI-OCT approach for biopsy guidance.

## 5. Conclusions

The use of a novel AI-OCT approach for analyzing tissue composition at the tip of the biopsy needle was analyzed. OCT was able to provide high-quality images of the tissue at the tip of the biopsy needle, while the cloud-based AI analysis of these images seemed to provide suitable results for analyzing tissue composition in real time. However, further improvements are still needed to make the technology able to provide more accurate results, which will likely improve its potential for clinical adoption. The use of large-sized training sets of images is deemed to be necessary. A human trial is planned to generate large training sets of images and further improve AI accuracy.

## Figures and Tables

**Table 1:** Definition of AI model classes and tracked features.

CNN	Definition	Excluded Features	Number of Annotated Images
**CNN1: Tissue**	All tissue including muscle, fat, vessel, and tumor.	Dark background, catheter, and tissue holes.	89
**CNN2: Tumor**	- Bright dense tumor region. - Darker region including clear features of muscle (fibrous) and fat (glittery).	- Regions away from catheter surface where signal fades. Bright and straight line (vessel). - Regions away from catheter surface where signal fades.	94
**CNN3: Necrotic** **Tumor**	Focal dark region surrounded by tumor region.	Dark regions away from catheter surface where signal fades.	72

**Table 2:** AI model training and augmentation parameters for each class.

		CNN1: Tissue	CNN2: Tumor	CNN3: Necrotic Tumor
**Training** **parameters**	Weight decay	0.0001	0.0001	0.000140
	Mini-batches size	20	20	20
	Mini-batches per iteration	40	20	40
	Iterations without progress	500	500	500
	Initial learning rate	1	1	1
**Image** **augmentation**	Scale (Min/max)	−1/1.01	−1/1.01	−1/1.01
	Aspect ratio	1	1	1
	Maximum shear	1	1	1
	Luminance (min/max)	−1/50	−1/1	−1/1
	Contrast (min/max)	−1/50	−1/50	−1/50
	Max with balance change	1	1	1
	Noise	0	0	0
	JPG compression (min/max)	40/60	40/60	40/60
	Blur max pixels	1	1	1
	JPG compression percentage	0.5	0.5	0.5
	Blur percentage	0.5	0.5	0.5
	Rotation angle (min/max)	180/180	−180/180	−180/180
	Gain	1	1.5	1.3
	Level of detail	Low	Medium	Medium

**Table 3:** Definition of AI model accuracy indices.

Parameter	Formula
**False Positive (FP) (%)**	This parameter determines the proportion of pixels incorrectly classified as positive in the verification region.
**False Negative (FN) (%)**	This parameter determines the proportion of pixels incorrectly classified as negative in the verification region.
**Error (%)**	(FP + FN)/All validation area
**Precision (%)**	TP/(TP + FP)
**Sensitivity (%)**	TP/TP + FN
**F1 Score (%)**	2TP/(2TP + FP + FN)

**Table 4:** Calculated accuracy parameters and human–AI model agreement as an average for all 4 tissue classes.

	FP %	FN %	Error %	Precision %	Sensitivity %	F1 Score %
**AI vs. Human**	0.86	1.81	2.67	73.17	66.98	77.36
**Human vs. Human**	1.53	1.47	3.00	71.23	71.23	76.74
**F1 score Agreement**	99.38%					

**Table 5:** Calculated accuracy parameters and human–AI model agreement for tumor regions.

	FP %	FN %	Error %	Precision %	Sensitivity %	F1 Score %
**AI vs. Human**	1.25	1.12	2.37	65.11	68.5	74.74
**Human vs. Human**	1.41	1.37	2.78	69.66	69.66	71.89
**F1 score Agreement**	97.15%					

**Table 6:** Calculated accuracy parameters and human–AI model agreement for normal tissue.

	FP %	FN %	Error %	Precision %	Sensitivity %	F1 Score %
**AI vs. Human**	1.14	1.21	2.35	77.36	73.15	76.45
**Human vs. Human**	1.39	1.26	2.64	73.96	73.96	78.48
**F1 score Agreement**	97.97%					

**Table 7:** Calculated accuracy parameters and human–AI model agreement for necrotic tissue regions.

	FP %	FN %	Error %	Precision %	Sensitivity %	F1 Score %
**AI vs. Human**	0.58	4.23	4.82	38.9	18.27	42.11
**Human vs. Human**	2.62	2.56	5.17	41.7	41.7	57.53
**F1 score Agreement**	84.58%					

## Data Availability

Data supporting the reported results are considered proprietary to Physical Sciences and cannot be released without signing a confidentiality agreement.
